# Technological Benefits Associated with the Use of Spray-Dried Animal Plasma in Fish-Based Chunks for Canned Pet Food

**DOI:** 10.3390/ani13223460

**Published:** 2023-11-09

**Authors:** María-Carmen Rodríguez, Ciro Cámara, Salvador Morera, Neus Saborido, Javier Polo

**Affiliations:** 1 R&D Department, APC Europe SLU, PI Congost, Av. San Julián 246-258, 08403 Granollers, Spain; neus.saborido@apc-europe.com (N.S.); javier.polo@apc-europe.com (J.P.); 2 Essentia Protein Solutions, Ulsnæs 33, DK-6300 Gråsten, Denmark; ciro.camara@essentiaproteins.com (C.C.); salvador.morera@essentiaproteins.com (S.M.)

**Keywords:** chunks in gravy, fish by-products, spray-dried animal plasma, texture, wheat gluten, wet pet food

## Abstract

**Simple Summary:**

Fish and fish by-products are excellent sources of high-quality protein for cats and dogs. However, the handling, processing, and storage of raw materials can increase variation and negatively impact their technological properties. This study aimed to evaluate the use of spray-dried animal plasma (SDAP) as a binder in fish by-products recipes for chunks in gravy. The results showed that the inclusion of SDAP in recipes significantly improved the consistency (hardness), elasticity (springiness), cohesiveness, chewiness, and juiciness of the final wet products. These improvements were observed in a chunk recipe with a 35% content of mixed salmon and tuna by-products, and another recipe with chunks having a final content of a 4% salmon by-product. There was a positive linear effect of increased SDAP inclusion in the recipes for most of the technological parameters measured such as elasticity, flexibility, juiciness, and hardness. Our findings indicate that SDAP is an excellent nutritional binder that can enhance the final technological properties of wet pet food products using high-quality fish recipes and potentially in recipes containing protein-rich fish by-products with low functionality.

**Abstract:**

Spray-dried animal plasma (SDAP) and wheat gluten (WG) are common binders in wet pet food that provide amino acids and energy, as well as texture and cohesiveness due to their gelling strength, water retention and fat emulsion properties. Binder use is a valuable tool especially in recipes based on ingredients with low technological properties such as fish by-products containing spines and scales and soft texture after cooking. Two basal recipes for chunks in gravy were produced to evaluate experimental treatments. One basal recipe used a mixture of salmon and tuna by-products as the only animal protein sources without binders or with a 20 g/kg inclusion of SDAP or WG. The other basal recipe mimicked a more typical commercial recipe containing meat animal ingredients and a 40 g/kg salmon by-product to develop experimental treatments with and 0, 10, 20, 30 or 40 g/kg inclusions of SDAP. Dry matter, protein, and viscosity were evaluated in raw emulsions. After a 1 h retorting at 121 °C, hardness was measured in emulsions and in cooked chunks, juiciness, and Texture Profile Analysis (TPA) were assessed. Results demonstrated the viability of producing quality chunks in gravy containing only fish by-products including 20 g/kg of SDAP, which significantly increased hardness, elasticity, cohesiveness, and juiciness. There was a positive linear correlation of increased SDAP inclusion rate in the commercial recipe for most of the quality parameters evaluated. Based on these results, the inclusion of SDAP in fish recipes can help manufacturers achieve technological quality control targets for commercial wet pet food and may help producers to successfully formulate new recipes for wet pet food products using fish by-products as the sole animal protein source.

## 1. Introduction

Many moist pet food products are packaged in metal cans. However, the use of flexible packaging (pouches) has become an attractive option in recent years, not only for the exceptional labeling graphics and marketing possibilities, but also because they offer tremendous energy savings both in their production and transport. Additionally, pouches are easier to open than cans; they weigh much less and can have a smaller environmental impact compared to cans. The use of pouches is growing, especially in Europe, due to their convenience, because this format involves putting food into single-serve containers, avoiding the necessity of keeping half-eaten tins in the refrigerator and ensuring that the food is always served fresh. Pouches are especially focused on cat food products since cats prefer aroma and texture, and single-serve formats ensure a fresh easy-to-serve meal each time at room temperature. However, flexible packaging is less physically protective against handling damage to the chunks during processing, transport, distribution and to the final consumer compared to canned products. The use of binders in these small chunks is essential to maintain the texture and cohesion of the final products. 

Spray-dried animal plasma (SDAP) is characterized by its high protein content, water-holding capacity, foaming and emulsifying properties [1,2], and gel strength when heated above 80 °C [3,4,5,6]. Each of these technological properties are highly appreciated in the food and pet food industries that produce cooked meat products for human or pet consumption.

The inclusion of SDAP in wet pet food recipes allows for better homogenization of the quality of the final cooked product independently of small differences between batches of raw meat by-product materials, or changes in composition related to the temporal availability of meat ingredients [7,8]. SDAP is one of the most common, useful, and appreciated binders in wet pet food products, especially for chunks in gravy and pouch-type products because it helps to bind ingredients, confers elasticity and cohesiveness to the chunks and provides visual benefits after cooking and cutting by reducing the formation of crumbs; it also benefits filling operations. It minimizes mechanical damages to the final product caused by handling, transportation, and storage throughout the distribution chain of the product. Overall, SDAP helps preserve chunk integrity in the finished product.

In addition to all the processing properties of SDAP in wet pet food, two recent reviews [9,10] summarized many other benefits associated with the use of SDAP in animal feed, including dog and cat food. Nutritionally, SDAP has a high protein concentration with an appropriate composition of amino acids, and it is highly digestible for dogs and cats [11,12,13]. It contains many bioactive compounds such as immunoglobulins, cytokines, growth factors, enzymes, and metalloproteins that confer immunomodulatory, anti-inflammatory, prebiotic and neuroprotective properties as demonstrated in animal models [14,15,16,17].

Fish is a good source of high-quality protein, and there are many advantages recognized in the literature [18,19] associated with the use of fish substrates as a protein source. Fish muscle contains iodine, and fish ground bones are a good source of calcium and phosphorous. The flesh of oily fish (herring, pilchards, mackerel, sardines, tuna, salmon, and trout) contains significant amounts of vitamins A and D and omega-3 fatty acids. In the last decade, research clearly showed that omega-3 fatty acids had many positive health effects on the immune system, skin and hair coat, cancer, and cognitive function in dogs and cats [20,21]. Omega-3 fatty acids associated with potential health benefits include docosahexanoic acid (DHA), eicosapentanoic (EPA) and alpha-linolenic acid (ALA). High concentrations of DHA and EPA can be found in cold-water fatty fish such as salmon, tuna, mackerel, herring, and sardines, whereas ALA is present in canola oil, flaxseed, and other ingredients. Although ALA can be converted to EPA and DHA, the metabolism of dogs and cats is not very efficient, and it is able to produce only limited amounts [22]. Therefore, several super-premium and premium foods have been reformulated with a fish source containing these fatty acids that can improve cognitive functions, memory and learning ability [19]. Fish oil is a commonly used supplement in pet food, and several studies report health benefits from fish oil supplementation in dogs and cats [23,24]. Many wet pet foods use fish sources to add variety to meals for dogs or cats, and fish is a frequent raw material used in cat pet food. Although fish is not the natural diet of cats, people believe that cats seem to be keen on eating fish [25]. Moreover, a high-level use of quality fish protein in products has led to the highlight of the inclusion of this source in the label as “with salmon” or “with tuna”.

However, fish by-products, which include whole fish or fish body parts from species suitable for human consumption, may exhibit several quality issues related to stability and rancidity. Furthermore, these fish by-products are characterized by low technological functionality including issues with texture, binding, and emulsifying properties. These limitations primarily stem from the absence of suitable processing operations that preserve functionality [26]. Additionally, these challenges can be attributed, in part, to the inherent nature of the meat, characterized by low connective tissue content [27,28], and, to some extent, to the variability in composition (heads, viscera, scales, skin, and skeleton) across species, seasons, and fishing locations.

Low technologically functional ingredients, such as fish, cause processing difficulties during the production of wet pet food products, especially in pouches containing chunks for cats. Recipes for chunks normally are formulated to meet all the dietary nutrients required by cats and include very small pieces of meat or fish-based food (chunks) imbibed in a liquid sauce (gravy) or in a semi-solid sauce (jelly). Ingredients are normally a mixture of meat or fish by-products, cereals, vitamins, and minerals. The integrity of chunks in pouches is extremely difficult to maintain throughout the production chain, which involves cooking, cutting, filling, final packaging, and commercial distribution. The low functionality of fish by-products is likely one of the reasons why the list of premium or super-premium products based exclusively on fish by-products is limited. Most of the fish products commercially available normally contain up to 4% fish by-products in the final product, although recently some products with higher (32%) fish by-product content can be found.

The aim of this study was to test the technological properties associated with the inclusion of SDAP in recipes for chunks in gravy containing frozen fish by-products and in recipes where fish by-products are the only protein source. We hypothesized that the inclusion of SDAP into fish-based chunks in gravy recipes would improve product texture, water binding, and fat emulsion, even though these ingredients have relatively low technological properties which can limit their use in certain wet pet food recipes.

## 2. Materials and Methods

### 2.1. Raw Materials

Canned pet food recipes (chunks in gravy) were formulated using three different batches of three different frozen animal by-products from a Spanish supplier to European wet pet food producers (Corsa PetFood, S.L., Barcelona, Spain). Frozen fish by-products (salmon or tuna) consisted of whole fish without viscera, or parts of fish with heads, bones, and tails. Chicken carcass, used as a control, was composed of ground chicken carcass with gizzards and liver in natural proportion, clean and without bile, and could also contain meat pieces, bones, necks, wings, and thighs. The main nutritional characteristics of these raw materials are summarized in Table 1.

A composite sample of the three lots of each animal by-product used was prepared and sent for fat analysis to an external official laboratory of analysis (Ofice S.L., Barcelona, Spain) to confirm the fat content of the technical data sheet.

Pet food diets were produced with or without binders. Two different binders were used, SDAP (AP820PF, APC-Europe S.L.U., Granollers, Spain) and WG (Viten, Roquette, Lestrem, France). The composition of both binders used is indicated in Table 2.

### 2.2. Formulation of Canned Pet Foods

In this study, two types of chunks in gravy at a 48:52 ratio were designed and used in two experiments. In all recipes, the gravy contained 8 g/kg of pork stock (Proliant P1301), 1.5 g/kg of iota carrageenan (Ceamvis 3383, CEAMSA, Porriño, Spain) and 990.5 g/kg of water.

In Experiment 1, both raw materials, chicken carcass and a 50:50 mixture of frozen salmon and tuna, were tested in a simple chunk recipe where they were the main ingredients (Table 3). Recipes were formulated with and without both binders (SDAP and WG) at 20 g/kg in the chunk recipe.

In Experiments 2 and 3, chicken carcass and salmon by-products were tested in a more typical commercial recipe for chunks containing several terrestrial meat by-product ingredients (Table 4). Frozen salmon by-product was tested instead of the mixture of salmon/tuna, since salmon is intrinsically a more difficult raw material to process due to its softer texture.

In Experiment 2, recipes containing a salmon by-product replacing chicken carcass in the control recipe were designed to have an inclusion of 40 g of salmon/kg in the final product including gravy. In Experiment 3, similar recipes without any binder or with increasing percentages of SDAP were tested.

The three experiments were replicated three times over three different days of production. All treatments were performed each day using the three different lots of animal by-products for statistical purposes. However, a single lot of binders was used for all experiments as indicated in Table 2.

### 2.3. Production of Diets

Frozen animal by-products were ground with a lab-scale meat grinder (Cato SA, Sabadell, Barcelona, Spain) fitted with a die plate with 3 mm diameter holes. After grinding, they were mixed with the different additives (salt, sodium polyphosphate, sodium bicarbonate, locus bean gum and part of the water) in a pilot plant meat bowl-chopper (Cato SA, Sabadell, Barcelona, Spain) at maximum speed (2600 rpm). After the initial mixing, wheat flour was added with the rest of water until a homogeneous blend was obtained, which required approximately 3 min. Finally, SDAP or WG were added to the meat bowl-chopper and the blending process was finished when the raw emulsion reached 12–14 °C.

For each dietary treatment and for each day of experiment, a sample of raw emulsion was analyzed for dry matter, protein, and ash content. Six Lacquer cans (400 g, diameter 72 mm, height 10.4 cm) were filled with the raw emulsion to measure viscosity, and eight additional cans were filled to a 1 cm headspace to measure hardness after retorting at 121 °C for 1 h in an Austester 437 G autoclave (J.P. Selecta, Abrera, Spain). Samples were left at room temperature for two weeks before the measurement of hardness was performed. 

Chunks of similar size and shape (35 × 17 × 30 mm^3^) were produced with a special proprietary device. After cooking the raw emulsion at 90 °C for 10 min in a Laint HCR steam oven (Laint, Abad maquinaria industrial, S.L., Sentmenat, Spain), the cooked emulsions were cooled in a refrigerator (4 °C) for 20 min before being canned. Four cans of each recipe were prepared containing 15 chunks previously weighed and embedded in gravy at a 48:52 chunks: gravy ratio. Cans were sterilized at 121 °C for 1 h, and they were left to cool at room temperature for two weeks before measurements.

In all cases after filling, cans were sealed with a manual round can seamer with an easy open lid before retorting. Cans of different experimental recipes were mixed and distributed randomly in the autoclave. Measurements were performed after two weeks of storage at room temperature from day of production to allow the interaction of the gravy with the chunks.

### 2.4. Nutritional and Viscosity Analyses

Water content was analyzed according to the AOAC (1999) procedure 930.15 [29]. Ash was determined according to the European official method [30]. Protein content was analyzed by the combustion method AOAC procedure 990.03 [30]. Fat was measured by gravimetry using an in-house method based on Regulation (EC) No.152/2009.

For each experiment, six cans were randomly selected to measure viscosity of the raw emulsions using a Brookfield DV-E viscosimeter (Brookfield Engineering Laboratories, Inc., Middleboro, MA, USA) with a spindle RV-7 set at 22–24 °C and speed at 100 rpm.

### 2.5. Analysis of Processed Canned Pet Foods

The hardness of the cooked emulsion was measured in eight cans of each recipe and day of experiment as described by Polo et al. in 2005 [5]. Briefly, raw emulsion was canned and sterilized at 121 °C for 1 h. Two weeks later, they were analyzed by penetration with a cylinder probe (P/0.5) with 120 mm of diameter and a 30 kg load cell using texture analyzer TA-XT2 (Stable Micro System, Surrey, UK). The probe was placed centrally in the can and pushed into the cooked emulsion at 0.5 mm/s. The penetration test (20 mm) started at 20 g of activation force. Hardness was recorded as the force needed to break the product, and it is expressed in Newtons.

Each day of production, a total of 20 chunks of each dietary treatment (5 chunks randomly selected from 4 cans produced per treatment) were analyzed by Texture Profile Analysis (TPA) with the TA-XT2i analyzer (Stable Micro System, Surrey, UK) according to procedures described by Polo, 2011 [8]. TPA is a common and useful method used for years to evaluate food texture, and it is well correlated with sensory parameters [31,32,33]. For this analysis, chunks of similar size (35 × 17 × 30 mm^3^) were produced with a special proprietary device. The analysis consists of a double compression cycle (“double bite”) of the sample with a time to bounce back in between 2 s. Both cycles were performed at the same constant speed of 0.5 mm/s and 50% of strain. Several textural parameters were obtained automatically (Table 5).

The juiciness or succulence of chunks was evaluated as the quantity of gravy absorbed by the chunk after two weeks of production, which corresponds to the change in weight experienced by chunks [34]. Chunks were first removed from the gravy, and with the help of an absorbent paper, the outside gravy was eliminated. The chunks were weighed, and the result was used to calculate juiciness as a percentage of gravy retained by chunks ((final weight − initial weight)/initial weight × 100).

### 2.6. Statistical Analyses

Results are presented as means of data collected on three different experimental production days. Dry matter, protein and ash were measured in a sample per recipe on each of the three days of production both in the raw emulsions and animal by-products used. Viscosity was measured in six cans by recipe on each day of production. The texture of raw emulsion was evaluated after retorting in eight cans from each day of production and recipe. Juiciness was evaluated in 4 cans of chunks in gravy by treatment and all TPA parameters in 20 chunks, 5 from each can.

All data were analyzed using the PROC general linear models of statistical analysis software (SAS version 9.4, SAS Institute Inc., Cary, NC, USA). For all experiments, treatment was the independent variable, and dependent variables were dry matter, protein, ash, viscosity, and hardness in emulsions, and juiciness, hardness, adhesiveness, springiness, cohesiveness, and chewiness in chunks.

In Experiments 1 and 2, data were subjected to analysis of variance (ANOVA) procedures for a completely randomized design to detect differences among treatments. The mean values of treatments were calculated using least squares means (LSMEANS). If treatment effects were found, least squares means were used to differentiate the treatments by the PDIFF option of SAS. Significance between differences was declared at *p* < 0.05 unless otherwise reported.

In Experiment 3, an analysis of variance for a completely randomized design with orthogonal contrasts for equally spaced treatments to determine linear, quadratic, cubic, and quartic effects of inclusion of SDAP in the formulas were utilized on the dependent variables. Treatment means were calculated using LSMEANS statement. Statistical significance was declared at *p* < 0.05 unless otherwise reported.

## 3. Results and Discussion

### 3.1. Raw Materials

Salmon had a significantly higher dry matter content than tuna and chicken carcass (Table 1). There were no significant differences in protein and ash among the three by-products used in wet pet food recipes. Even though fat content was not statistically compared, the fat content in salmon (18.04%) and chicken carcass (19.59%) was numerically higher compared to tuna (6.40%), which normally has a higher level of muscle and less fat content [20].

Wheat gluten had numerically higher protein and fat contents than SDAP and lower ash content (Table 2). The higher ash content in SDAP is attributed to the intrinsic minerals in blood to maintain the body osmotic pressure and to the residual anticoagulant added to blood to avoid coagulation during collection and processing [10].

### 3.2. Experiment 1

Results of hardness after retorting (121 °C 1 h), viscosity and nutritional composition of the emulsion with the mixture of salmon and tuna by-products with or without binders, and with chicken carcass without binders, are presented in Table 6. There were no significant differences in dry matter, protein, ash, and viscosity among raw emulsions. Despite the lack of statistical significance, fish by-product recipes with or without binders had numerically greater dry matter content than the chicken carcass recipe, and the inclusion of binders numerically increased the viscosity of the raw emulsion.

After retorting at 121 °C for 1 h, the hardness of the cooked emulsion was significantly lower in the recipe containing fish by-products without a binder compared to the recipe containing chicken carcass (25% reduction), as expected due to the low technological functionality of fish by-product (Table 6). The inclusion of wheat gluten at 20 g/kg to the recipe containing fish by-product did not significantly improve the hardness after cooking. However, the inclusion of SDAP at 20 g/kg to the fish by-product recipe resulted in significantly higher hardness than the control chicken carcass and the other fish by-products recipes. SDAP in the fish by-product recipe increased the final firmness of the product by 66% compared with the case when no binder was added, and by 58% with the same inclusion of wheat gluten. Polo et al. [5,7] also described similar improvements in texture using SDAP compared with WG in loaf and chunk recipes with inclusions of both binders at 15, 20 and 25 g/kg.

This increase in hardness obtained with the use of SDAP is probably related to its high gel strength capacity after heat treatment [4] and most likely to albumin. Albumin represents 45–55% of the plasma protein and it is the most heat-resistant protein that may be responsible for the improved texture obtained when SDAP is exposed to high temperatures [5]. Higher hardness after retorting reduces development of crumbs during the cutting process and throughout the handling, transport and can filling process (internal data). Higher resistance to crumb development due to better hardness of the cooked emulsion with SDAP may improve production yields and final quality appearance of the product. In contrast, hardness is not statistically different when WG is included compared with the fish recipe without binder.

Final chunks containing fish by-products after being imbibed in gravy for two weeks had significantly lower physical properties compared to those containing chicken carcass at the same inclusion level (Table 7) like the hardness results observed for the cooked emulsions. In general, fish chunks showed significantly lower values of consistency (hardness), elasticity (springiness), cohesiveness, and chewiness than chicken chunks. Fish chunks were easily disaggregated throughout the production chain, before being mixed with gravy and especially after the two weeks of storage in gravy since they became softer and were easily broken. During the production of fish chunks, there were numerous losses of product with many production difficulties such as the removal of unacceptable quality chunks from the conveyor belt, the formation of crumbs during cutting and losses of material during the manipulation for canning.

The inclusion of 20 g/kg of SDAP to the fish by-product recipe allowed a general and significant improvement in all the quality parameters measured (except for adhesiveness) in final fish chunks compared to those obtained with fish or fish with WG, and chicken by-products without any binder inclusion (Table 7). Chunks produced with fish containing 20 g/kg of SDAP showed the highest hardness, elasticity, cohesiveness, and chewiness. These results were consistent with those obtained by Polo [8] comparing different qualities of chicken carcass with different technological functionalities, and with internal data (not published) working with low-functional ingredients in pouches such as neck trims. These beneficial changes in physical technological quality derived from the addition of SDAP to the recipe can maintain desired chunk integrity during production, manipulation, and transport before and after being mixed with the gravy, and after two weeks of storage in cans.

The use of 20 g/kg of WG in the fish recipe did not significantly modify any of the quality parameters measured (Table 7) in fish chunks, although after cooking, they showed a little more numerical consistency (hardness) and chewiness than the chunks produced without binders, and lesser losses of product were observed during production (not measured). In general, all the TPA values measured for the WG recipe were like those of the salmon and tuna by-product recipes without binders.

Fish by-product chunks without binders showed higher juiciness compared to those of chicken carcass, probably because fish by-products contain higher levels of fat than chicken and softer meat texture due to its low connective tissue content [27]. However, overall, the consistency of fish chunks was lower than that of chicken, and they did not maintain shape and structure and broke easily. Chunks produced with fish by-products and 20 g/kg of SDAP had the highest juiciness related to the absorption of gravy inside the chunk, which is released during mastication, without losing cohesiveness or integrity, and maintaining all its quality characteristics (Table 7). The inclusion of wheat gluten did not improve the juiciness of fish chunks. Similar results were also obtained by Polo and Rodriguez [34] working with meat chunks with different inclusions of WG compared to a 20 g/kg inclusion of SDAP. The authors related this higher juiciness with improvements in palatability [5].

In conclusion, inadequate texture of fish chunks with or without 20 g/kg of WG did not maintain a desired final chunk integrity because these recipes have an increased presence of small crumbs generated throughout the various steps of emulsion cooking, cutting, can filling and post-retort in cans because they did not show an adequate chunk consistency and the ingredients were not properly bound after cooking. However, the use of 20 g/kg of SDAP in the same recipe allowed for production of fish chunks with higher physical qualities than that in the recipe using chicken carcass without a binder. Results demonstrated that SDAP is a good binder in wet pet food recipes and is superior to WG in the fish-based recipes tested. Others have reported a similar conclusion for loaf recipes containing meat ingredients with different binders, including WG and SDAP, and for recipes that had excess fat or water [6,7].

### 3.3. Experiments 2 and 3

The raw emulsion of the commercial recipe containing 85 g/kg of salmon by-product without a binder had numerically lower dry matter (*p* = 0.0692), protein (*p* = 0.2219) and viscosity (*p* = 0.3786), and significantly lower ash (*p* = 0.0263) contents before cooking. Retorting at 121 °C for 1 h produced a significantly lower emulsion hardness compared to those of the chicken recipe (Table 8).

Chunks containing chicken or salmon without binder did not differ significantly for most TPA parameters, but the salmon recipe had significantly lower hardness and chewiness in the chunks after cooking and storage at room temperature for two weeks. In contrast to Experiment 1 results, the juiciness of the salmon recipe was similar to that of the chicken one, although it was numerically lower (*p* = 0.1267) in Experiment 2, and this result may be due to the much lower percentage of fish by-product inclusion used in Experiment 2 recipes compared with those in Experiment 1.

**Figure 1:**
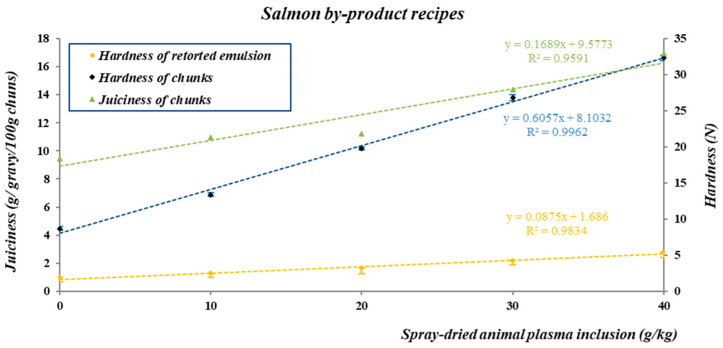
Correlation and prediction equations for the relationship of spray-dried plasma inclusion and hardness of the retorted emulsion (121 °C, 1 h) and juiciness and hardness of chunks produced with recipes from Experiment 3.

In Table 9, results from Experiment 3 show that as the amount of SDAP increased in the salmon recipe, dry matter, protein content, and viscosity of the raw emulsion increased linearly. The increased viscosity of the raw emulsion with increased inclusion of SDAP may reduce possible losses of the product during handling or pumping to the cooker. The hardness of the emulsion after retorting for 1 h at 121 °C increased linearly with the increasing inclusion of SDAP in the recipe, and there was a high correlation (R^2^ = 0.983) between SDAP inclusion and hardness of the retorted emulsion (Figure 1).

The use of SDAP in salmon by-product chunks linearly increased (*p* < 0.02) all TPA parameters as the inclusion of SDAP increased from 0 to 40 g/kg of SDAP (Table 10). As observed for hardness of the retorted emulsion, there was a high correlation between increased inclusion of SDAP and increased hardness of chunks (R^2^ = 0.996) and juiciness of chunks (R^2^ = 0.959) as shown in Figure 1. Although not shown in Figure 1, correlation coefficients between the inclusion of SDAP and adhesiveness (R^2^ = 0.956), springiness (R^2^ = 0.916), cohesiveness (R^2^ = 0.863) and chewiness (R^2^ = 0.982) of chunks were also very high.

The high correlations between increased TPA parameters and the increased inclusion of SDAP in recipes can be highly valuable for pet food producers. This correlation allows them anticipation of the physical product quality, encompassing attributes like hardness, flexibility, and cohesiveness, for example, even in scenarios where recipe adjustments are necessitated by factors such as ingredient availability or variations in ingredient technological quality. Manufacturers can meet their specific technical quality standards by optimizing the percentage of SDAP in alignment with their specific objectives. Moreover, this correlation facilitates the prediction of the final product quality across diverse product presentations, including variations in chunk size and packaging options.

Furthermore, it was interesting to see that the chunk hardness results in Experiment 1 for the recipe using 20 g/kg of SDAP and fish by-products (salmon and tuna) as the only source of animal protein were quite similar to the values obtained in Experiment 3 with the recipe containing 30 g/kg of SDAP and 85 g/kg of salmon by-product (27.9 N vs. 26.84 N per respective experiment). In fact, chunk hardness values from Experiments 1 and 3 were not significantly different by Student *t*-test (*p* = 0.126). The consistency of chunk hardness results between experiments suggest that it may be possible to produce small chunks for pouches containing only fish proteins without the use of other meat animal by-product proteins when SDAP is included as a binder.

More research is needed to further clarify an ideal or recommended percentage of inclusion of SDAP in this kind of fish wet pet food products using only fish protein products. Future experiments using commercial formats in terms of chunk size, packaging and real retorting conditions would be needed to confirm the improvements described in these experiments. Palatability is closely related to texture (chewiness, hardness, springiness) of pet food products. Recipes for both dogs and cats with different inclusion rates of SDAP using different fish and fish by-products other than those of salmon origin should be submitted to palatability tests that also compare palatability with recipes containing other meat animal by-products with better technological properties.

## 4. Conclusions

Results indicated that the inclusion of spray-dried animal plasma in chunks in gravy could provide pet food manufacturers a technological solution to produce any kind of fish-based products, even when fish is used at a low inclusion level, or when fish is the only animal protein by-product present in the formulation. SDAP significantly improved most of the parameters that defined texture or consistency such as hardness, chewiness, elasticity, or cohesion after retorting. The increased inclusion of SDAP in a recipe for chunks in gravy using primarily salmon by-product was highly correlated with improvements in texture and juiciness defined as the quantity of liquid released when an animal chews the chunk.

## Figures and Tables

**Table 1:** Nutritional composition of the three different animal by-products used in chunks in gravy pet food recipes ^1^.

Parameter	Chicken ^2^	Tuna ^3^	Salmon ^4^	*p*-Value
Dry matter (%)	33.3 ± 0.31 ^b^	28.96 ± 0.69 ^a^	37.73 ± 0.23 ^c^	<0.001
Protein (%)	14.6 ± 0.71	14.85 ± 0.31	13.44 ± 0.91	0.234
Ash (%)	3.53 ± 0.37	3.54 ± 0.05	3.51 ± 0.30	0.749
Fat ^5^ (%)	13.00	7.00	17.00	---

^1^ Results are expressed as mean ± standard error. ^2^ Chicken carcass was composed of ground chicken carcass with gizzards and liver, and could contain meat pieces, bones, necks, wings, and thighs. ^3^ Tuna by-product consisted of whole frozen tuna without viscera or parts of fish with heads, bones, and tails. ^4^ Salmon by-product contained whole frozen salmon without viscera or parts of fish with heads, bones, and tails. ^5^ Fat content according to the technical data sheet of each animal by-product from Corsa Pet Food S.L. (Barcelona, Spain). ^abc^ Values with different superscripts in the same row are significantly different by Multiple Sample Comparison.

**Table 2:** Nutritional composition of the binders used in chunks in gravy recipes.

Binder	Spray-Dried Animal Plasma ^1^	Wheat Gluten ^2^
Dry matter (%)	91.02	90.47
Protein (%)	72.53	80.05
Ash (%)	14.58	1.11
Fat (%)	2.19	5.77

^1^ Spray-dried animal plasma is a protein product in powder form composed of albumin and globulin proteins with their biological characteristics preserved. ^2^ Wheat gluten is a protein fraction in powder form extracted from wheat by wet milling.

**Table 3:** Recipe composition of chunks produced in Experiment 1.

Ingredients (g/kg)	Chicken	Fish	Fish + 20 g/kg SDAP	Fish + 20 g/kg WG
Chicken carcass ^1^	711.9	0	0	0
Fish by-product ^2^	0	711.9	691.9	691.9
Wheat flour	70.0	70.0	70.0	70.0
SDAP ^3^	0	0	20.0	0
WG ^4^	0	0	0	20.0
Locus bean gum	5.0	5.0	5.0	5.0
Common salt	5.0	5.0	5.0	5.0
Sodium polyphosphate	5.0	5.0	5.0	5.0
Sodium bicarbonate	2.0	2.0	2.0	2.0
Ascorbic acid	1.0	1.0	1.0	1.0
Sodium nitrite	0.1	0.1	0.1	0.1
Water	200	200	200	200
Gravy	1083	1083	1083	1083
TOTAL	2083	2083	2083	2083

^1^ Chicken carcass was composed of ground chicken carcass with gizzards and liver and could include meat pieces, bones, necks, wings, and thighs. ^2^ Fish by-product was a homogeneous mixture of frozen salmon and tuna by-products at a 50:50 ration by weight (CorsaPet Food S.L., Barcelona, Spain). ^3^ SDAP: spray-dried animal plasma (AP820PF from APC Europe S.L.U., Granollers, Spain). ^4^ WG: wheat gluten (Viten from Roquette, Lestrem, France).

**Table 4:** Recipe composition of chunks produced in Experiment 2 containing 40 g/kg of the final product of salmon by-product with different percentages of spray-dried animal plasma (SDAP) compared with a control recipe with chicken carcass and without SDAP.

Ingredients (g/kg)	Control Group (Chicken)	Experimental Group (Salmon)				
		0 g/kg SDAP	10 g/kg SDAP	20 g/kg SDAP	30 g/kg SDAP	40 g/kg SDAP
Chicken carcass ^1^	411.0	326.0	316.0	306.0	296.0	286.0
Salmon by-product ^2^	0	85.0	85.0	85.0	85.0	85.0
Pig lungs and trachea	186.0	186.0	186.0	186.0	186.0	186.0
Chicken necks	92.0	92.0	92.0	92.0	92.0	92.0
Pig liver	20.0	20.0	20.0	20.0	20.0	20.0
Wheat flour	60.0	60.0	60.0	60.0	60.0	60.0
SDAP ^3^	0	0	10.0	20.0	30.0	40.0
Locust bean gum	4.4	4.4	4.4	4.4	4.4	4.4
Common salt	5.0	5.0	5.0	5.0	5.0	5.0
Sodium polyphosphate	5.0	5.0	5.0	5.0	5.0	5.0
Sodium bicarbonate	1.6	1.6	1.6	1.6	1.6	1.6
Water	215	215	215	215	215	215
Gravy	1083	1083	1083	1083	1083	1083
Total	2083	2083	2083	2083	2083	2083

^1^ Chicken carcass was composed of ground chicken carcass with gizzards and liver, and could include meat pieces, bones, necks, wings, and thighs. ^2^ Frozen salmon by-product contained whole salmon without viscera or parts of fish with heads, bones, and tails; ^3^ SDAP: spray-dried animal plasma (AP820PF from APC Europe S.L.U., Granollers, Spain).

**Table 5:** Parameters, units, and definition of the main variables derived from the Texture Profile Analysis (TPA) curve.

Parameter	Measured Variable	Units	Definition
Adhesiveness	Work	N·s	Negative force area of the first compression cycle. It is the work necessary to pull the plunger away from the sample.
Chewiness	Work	N·mm	Product of hardness times cohesiveness times springiness. Only applied to solid products.
Cohesiveness	Ratio of forces	dimensional	Ratio of the positive force area during the second compression cycle to that during the first cycle.
Hardness	Force	N	Peak force during the first compression cycle.
Springiness	Distance	mm	Length to which the sample recovers in height during the time elapsed between the end of the first compression cycle and the start of the second cycle.

**Table 6:** Nutritional composition and viscosity of the raw emulsions used in chunks of Experiment 1 and hardness values after retorting at 121 °C for 1 h ^1^.

	Chicken ^2^	Fish ^3^	Fish + 20 g/kg SDAP ^4^	Fish + 20 g/kg WG ^5^	SEM ^6^
Dry matter (%)	31.48	32.02	32.59	32.98	0.45
Protein (%)	10.57	10.58	11.90	10.63	0.45
Ash (%)	3.40	3.68	3.64	3.17	0.45
Viscosity (Pa.s)	57.10	56.29	59.29	59.02	4.78
Hardness (N) ^7^	3.32 ^b^	2.49 ^a^	4.12 ^c^	2.60 ^a^	0.16

^1^ Results are expressed as mean ± standard deviation. ^2^ Chicken carcass was composed of ground chicken carcass with gizzards and liver, and could include meat pieces, bones, necks, wings, and thighs. ^3^ Fish by-product is a homogeneous mixture of frozen salmon and tuna by-products at a 50:50 ratio by weight (CorsaPet Food S.L., Barcelona, Spain). ^4^ SDAP: spray-dried animal plasma (AP820PF from APC Europe S.L.U., Granollers, Spain). ^5^ WG: wheat gluten (Viten from Roquette, Lestrem, France). ^6^ SEM is the standard error of the mean. ^7^ Hardness was measured after cooking raw emulsions at 121 °C for 1 h in cans. ^abc^ Values with different superscripts in the same row were significantly different (*p* < 0.05).

**Table 7:** Juiciness and Texture Profile Analysis (TPA) of chunks in gravy produced in Experiment 1 after two weeks of storage at room temperature ^1^.

	Chicken ^2^	Fish ^3^	Fish + 20 g/kg SDAP ^4^	Fish + 20 g/kg WG ^5^	SEM ^6^
Juiciness ^7^ (%)	11.64 ^a^	20.59 ^b^	23.97 ^d^	21.56 ^c^	0.26
Hardness (N)	15.53 ^b^	10.48 ^a^	27.92 ^c^	12.15 ^ab^	1.50
Adhesiveness (N.s)	−1.42 ^a^	−2.45 ^b^	−3.10 ^b^	−3.02 ^b^	0.20
Springiness (mm)	0.77 ^b^	0.63 ^a^	0.85 ^c^	0.63 ^a^	0.02
Cohesiveness	0.45 ^b^	0.34 ^a^	0.55 ^c^	0.33 ^a^	0.02
Chewiness (N.mm)	5.49 ^a^	2.28 ^a^	13.17 ^b^	2.57 ^a^	1.01

^1^ Results are means of three replicates by treatment recipe. ^2^ Chicken carcass was composed of ground chicken carcass with gizzards and liver, and could include meat pieces, bones, necks, wings, and thighs. ^3^ Fish by-product was a homogeneous mixture of frozen salmon and tuna by-products at a 50:50 ratio by weight (CorsaPet Food S.L., Barcelona, Spain). ^4^ SDAP: spray-dried animal plasma (AP820PF from APC Europe S.L.U., Granollers, Spain). ^5^ WG: wheat gluten (Viten from Roquette, Lestrem, France). ^6^ SEM is the standard error of the mean. ^7^ Juiciness represents the succulence of chunks or the percentage of gravy retained in chunks after two weeks of storage at room temperature as measured by difference of weight. ^abcd^ Values with different superscripts in the same row were significantly different (*p* < 0.05).

**Table 8:** Nutritional composition, viscosity, hardness of cooking emulsion, juiciness and TPA values of chunks in gravy from Experiment 2 ^1^.

	Chicken ^2^	Salmon ^3^	SEM ^4^	*p*-Value ^5^
Emulsion				
Dry matter (%)	31.48	29.37	0.60	0.0692
Protein (%)	10.57	10.12	0.22	0.2219
Ash (%)	3.40	2.66	0.15	0.0263
Viscosity (Pa·s)	57.10	51.61	3.93	0.3786
Hardness (N)	3.32	1.88	0.13	0.0013
Chunks in gravy				
Juiciness ^6^ (%)	11.64	9.45	0.81	0.1267
Hardness (N)	15.18	8.72	0.76	0.0038
Adhesiveness (N·s)	−1.53	−0.99	0.16	0.0727
Springiness (mm)	0.76	0.65	0.03	0.0503
Cohesiveness	0.43	0.37	0.02	0.1392
Chewiness (N·mm)	5.00	2.17	0.40	0.0077

^1^ Results are means of three replicates by treatment. ^2^ Chicken carcass was composed of ground chicken carcass with gizzards and liver and could include meat pieces, bones, necks, wings, and thighs. ^3^ Salmon by-product contained whole frozen salmon without viscera or parts of salmon with heads, bones, and tails (CorsaPet Food S.L., Barcelona, Spain). ^4^ SEM is the standard error of the mean. ^5^ Differences were consider significant when *p* < 0.05. ^6^ Juiciness represents the succulence of chunks or the percentage of gravy retained in chunks after two weeks of storage at room temperature as measured by difference of weight.

**Table 9:** Nutritient composition of raw emulsions from Experiment 3, viscosity and emulsion hardness after retorting at 121 °C for 1 h ^1^.

	Dry Matter (%)	Protein (%)	Ash (%)	Viscosity (Pa·s)	Hardness (N)
0 g/kg SDAP ^2^	29.37	10.12	2.66	51.61	1.88
10 g/kg SDAP	29.70	10.71	2.86	52.98	2.46
20 g/kg SDAP	30.39	11.03	2.65	53.72	3.19
30 g/kg SDAP	31.01	11.40	2.59	55.24	4.29
40 g/kg SDAP	30.85	12.45	2.90	55.68	5.36
SEM ^3^	0.53	0.17	0.12	0.44	0.13
*p*-Value					
Linear	0.0243	<0.0001	0.5948	<0.0001	<0.0001
Quadratic	0.6064	0.1546	0.4099	0.5228	0.0176

^1^ Results are means of three replicates per recipe. ^2^ SDAP: spray-dried animal plasma (AP820PF from APC Europe S.L.U., Granollers, Spain). ^3^ SEM is the standard error of the mean.

**Table 10:** Juiciness and Texture Profile Analysis (TPA) of chunks in gravy produced in Experiment 3 after two weeks of storage at room temperature ^1^.

Recipes	Juiciness ^2^ (%)	Hardness (N)	Adhesiveness (N·s)	Springiness (mm)	Cohesiveness	Chewiness (N·mm)
0 g/kg SDAP ^3^	9.45	8.72	−0.98	0.65	0.37	2.17
10 g/kg SDAP	10.98	13.41	−0.76	0.76	0.45	4.62
20 g/kg SDAP	11.21	19.82	−0.99	0.88	0.69	10.59
30 g/kg SDAP	14.36	26.84	−0.52	0.93	0.69	17.33
40 g/kg SDAP	16.93	32.30	−0.49	0.95	0.74	22.60
SEM ^4^	1.25	0.76	13.4	0.02	0.05	0.70
*p*-Value						
Linear	<0.0001	<0.0001	0.0162	<0.0001	<0.0001	<0.0001
Quadratic	0.3122	0.4706	0.5553	0.0047	0.1054	0.0336

^1^ Results are means of three replicates by treatment. ^2^ Juiciness represents the succulence of chunks (percentage of gravy retained in chunks after two weeks of storage at room temperature and measured by difference of weight). ^3^ SDAP: spray-dried animal plasma (AP820PF from APC Europe S.L.U., Granollers, Spain). ^4^ SEM is the standard error of the mean.

## Data Availability

Data are available on reasonable request to the corresponding author.

## References

[1] Tybor P., Dill C., Landmann W. (1975). Functional properties of proteins isolated from bovine by a continuous pilot process. J. Food Sci..

[2] Etheridge P., Hickson D., Young C., Landmann W., Dill C. (1981). Functional and chemical characteristics of bovine plasma proteins isolated as a metaphosphate complex. J. Food Sci..

[3] Howell N., Lawrie R. (1984). Functional aspects of blood plasma proteins. II Gelling properties. J. Food Tech..

[4] Pares D., Saguer E., Saurina J., Suñol J.J., Carretero C. (1998). Functional properties of heat induced gels from liquid and spray-dried porcine blood plasma as influenced by pH. J. Food Sci..

[5] Polo J., Rodriguez C., Saborido N., Rodenas J. (2005). Functional properties of spray-dried animal plasma in canned petfood. Anim. Feed Sci. Technol..

[6] Polo J., Rodriguez C., Rodenas J., Morera S., Saborido N. (2007). Use of spray-dried animal plasma in canned chunk recipes containing excess of added water or chicken fat. Anim. Feed Sci. Technol..

[7] Polo J., Rodriguez C., Rodenas J., Morera S., Saborido N. (2009). The use of spray-dried animal plasma in comparison with other binders in canned pet food recipes. Anim. Feed Sci. Technol..

[8] Polo J. (2011). Standardizing Quality in Wet Pet food with Plasma.

[9] Vasconcellos R.S., Henriquez L.B.F., Lourenço P.d.S. (2023). Spray-Dried Animal Plasma as a Multifaceted Ingredient in Pet Food. Animals.

[10] Kazimierska K., Biel W. (2023). Chemical composition and functional properties of spray-dried animal plasma and its contribution to livestock and pet health: A review. Animals.

[11] Quigley J.D., Campbell J.M., Polo J., Russell L.E. (2004). Effects of spray-dried animal plasma on intake and apparent digestibility in dogs. J. Anim. Sci..

[12] Rodriguez C., Saborido N., Rodenas J., Polo J. (2016). Effects of spray-dried animal plasma on food intake and apparent nutrient digestibility by cats when added to a wet pet food recipe. Anim. Feed. Sci. Tech..

[13] de Andrade T., de Lima D.C., Domingues L.P., Felix A.P., de Oliveira S.G., Maiorka A. (2019). Spray-dried porcine plasma in dog foods: Implication on digestibility, palatability and haematology. Semin. Cienc. Agrar..

[14] Miró L., Garcia-Just A., Amat C., Polo J., Moretó M., Pérez-Bosque A. (2017). Dietary animal plasma proteins improve the intestinal immune response in senescent mice. Nutrients.

[15] Balan P., Staincliffe M., Moughan P.J. (2021). Effects of spray-dried animal plasma on the growth performance of weaned piglets—A review. J. Anim. Phys. Anim. Nutr..

[16] Moretó M., Miró L., Amat C., Polo J., Manichanh C., Pérez-Bosque A. (2020). Dietary supplementation with spray-dried porcine plasma has prebiotic effects on gut microbiota in mice. Sci. Rep..

[17] Garcia-Just A., Miró L., Pérez-Bosque A., Amat C., Polo J., Pallàs M., Griñán-Ferré C., Moretó M. (2020). Dietary spray-dried porcine plasma prevents cognitive decline in senescent mice and reduces neuroinflammation and oxidative stress. J. Nutr..

[18] Willard T. Utilization of marine by-products in pet foods. Proceedings of the International Conference of Fish By-Products.

[19] Zicker S.C., Jewell D.E., Yamka R.M., Milgram N.W. (2012). Evaluation of cognitive learning, memory, psychomotor, immunologic, and retinal functions in healthy puppies fed foods fortified with docosahexaenoic acid-rich fish oil from 8 to 52 weeks of age. J. Am. Vet. Med. Assoc..

[20] Biagi G., Mordenti A.L., Cocchi M., Mordenti A. (2004). The role of dietary omega-3 and omega-6 essential fatty acids in the nutrition of dogs and cats: A review. Prog. Nutr..

[21] Dominguez T.E., Kaur K., Burri L. (2021). Enhanced omega-3 index after long-versus short-chain omega-3 fatty acid supplementation in dogs. Vet. Med. Sci..

[22] Bauer J.E. (2008). Essential fatty acid metabolism in dogs and cats. R. Bras. Zootec..

[23] Hielm-Bjorkman A., Roine J., Elo K., Lappalainen A., Junnila J., Laitinen-Vapaavuori O. (2012). An un-commissioned randomized, placebo controlled double-blind study to test the effect of deep-sea fish oil as a pain reliever for dogs suffering from canine OA. BMC Vet. Res..

[24] Park H.J., Park J.S., Hayek M.G., Reinhart G.A., Chew B.P. (2011). Dietary fish oil and flaxseed oil suppress inflammation and immunity in cats. Vet. Immunol. Immunopathol..

[25] Houpy K.A., Smith S.L. (1981). Taste preferences and their relation to obesity in dogs and cats. Can. Vet. J..

[26] Folador J.F., Karr-Lilienthal L.K., Parsons C.M., Bauer L.L., Utterback P.L., Schasteen C.S., Bechtel P.J., Fahey G.C. (2006). Fish meals, fish components, and fish hydrolysates as potential ingredients in pet foods. J. Anim. Sci..

[27] Dunajski E. (2007). Texture of fish muscle. J Text. Stud..

[28] Rustad T. (2007). Physical and chemical properties of protein seafood by-products. Maximising the Value of Marine by-Products.

[29] (1999). Official Methods of Analysis of AOAC International.

[30] (1971). Official Journal of the European Union, First Commission Directive 71/250/EEC (1971).

[31] Bourne M.C. (1968). Texture profile of ripening pears. J. Food Sci..

[32] Friedman H.H., Whitney J.E., Szczesnial A.S. (1963). The texturometer—A new instrument for objective texture measurement. J. Food Sci..

[33] Meullenet J., Lyon B.G., Carpenter J.A., Lyon C.E. (1998). Relationship between sensory and instrumental texture profile attributes. J. Sens. Stud..

[34] Polo J., Rodriguez C. (2013). Improving the Juiciness of Pet Food Chunks and Pouches with Plasma.

